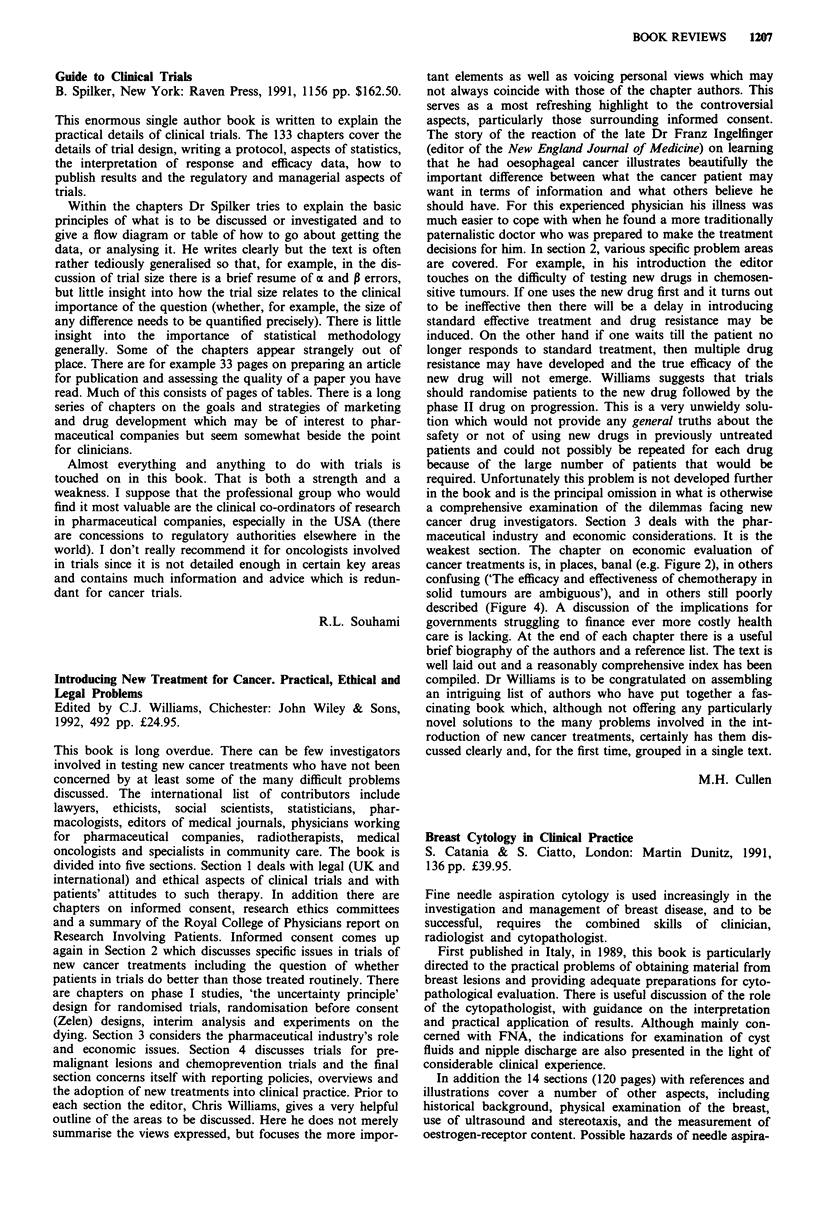# Guide to Clinical Trials

**Published:** 1992-12

**Authors:** R.L. Souhami


**Guide to Clinical Trials**
B. Spilker, New York: Raven Press, 1991, 1156 pp. $162.50.

This enormous single author book is written to explain the practical details of clinical trials. The 133 chapters cover the details of trial design, writing a protocol, aspects of statistics, the interpretation of response and efficacy data, how to publish results and the regulatory and managerial aspects of trials.

Within the chapters Dr Spilker tries to explain the basic principles of what is to be discussed or investigated and to give a flow diagram or table of how to go about getting the data, or analysing it. He writes clearly but the text is often rather tediously generalised so that, for example, in the discussion of trial size there is a brief resume of $\alpha$ and $\beta$ errors, but little insight into how the trial size relates to the clinical importance of the question (whether, for example, the size of any difference needs to be quantified precisely). There is little insight into the importance of statistical methodology generally. Some of the chapters appear strangely out of place. There are for example 33 pages on preparing an article for publication and assessing the quality of a paper you have read. Much of this consists of pages of tables. There is a long series of chapters on the goals and strategies of marketing and drug development which may be of interest to pharmaceutical companies but seem somewhat beside the point for clinicians.

Almost everything and anything to do with trials is touched on in this book. That is both a strength and a weakness. I suppose that the professional group who would find it most valuable are the clinical co-ordinators of research in pharmaceutical companies, especially in the USA (there are concessions to regulatory authorities elsewhere in the world). I don't really recommend it for oncologists involved in trials since it is not detailed enough in certain key areas and contains much information and advice which is redundant for cancer trials.

R.L. Souhami